# Influence of Personality and Motivation on Oral Presentation Performance

**DOI:** 10.1007/s10936-017-9551-6

**Published:** 2018-01-19

**Authors:** Hsin-Yi Liang, Brent Kelsen

**Affiliations:** 1 Department of Medicine, Chang Gung University, Taoyuan, Taiwan, ROC; 2 Department of Child Psychiatry, Chang Gung Memorial Hospital at Taoyuan, Taoyuan, Taiwan, ROC; 3 Language Center, College of Humanities, National Taipei University, 151 DaXue Rd, SanXia, New Taipei City, 237 Taiwan, ROC

**Keywords:** Extraversion, Big Five personality traits, Motivation, Collaborative Inquiry-based Project Questionnaire (CIPQ)

## Abstract

Personality and motivation have been identified as influential variables associated with foreign language learning; however, few studies have investigated their effect on oral presentations. This study addresses the importance of both personality and motivation in students’ collaborative oral presentation performance. A Big Five personality trait questionnaire measuring Extraversion, Agreeableness, Conscientiousness, Neuroticism and Openness to Experience, together with the Collaborative Inquiry-based Project Questionnaire measuring Task, Project Work, Reinforcement, Social Learning and Social Pressure motivational constructs were employed to evaluate 257 university students. In general, the results showed that Extraversion, Project Work and Social Pressure were significant correlates of oral presentation scores. The first result suggests that extraverts possess superiority in situations where oral language production is central to communication. This was particularly true for lower-level students, inferring that extraverted personalities can compensate for a lower English language ability. The second indicates that the inquiry-based nature of the assignments was an intrinsic motivator especially valued by extraverts. The third implies that extrinsic motivation was a factor influencing student performance. These findings extend previous research by highlighting the contextual relationships between these affective variables and performance in collaborative oral presentation contexts.

## Introduction

Personality research has shown that every individual possesses distinct combinations of personality characteristics that affect their emotions, perceptions, feelings, thoughts and motivations. Moreover, language learning success has been associated with individual personality and motivation differences (Dörnyei 1998; Dörnyei and Ushioda 2013; Dweck and Leggett 1988; Ehrman et al. 2003). In particular, extraversion has been recognized as a factor connected to successful language acquisition (Dewaele and Furnham 1999), and empirical research on foreign language performance and personality traits has revealed extraversion to be a significant variable of interest for language learning performance (Brown et al. 2001; Busch 1982; Ockey 2011).

Extraverts’ superiority in speech production is hypothesized to stem from their cognitive functioning, i.e. their ability to readily access short-term memory, perform better under stress and suffer less from bouts of anxiety (Dewaele and Furnham 1999; Howarth and Eysenck 1968). Dewaele and Furnham (1999) surmise that the advantages an extraverted personality offers a language learner are unlikely to be displayed in the production of written texts or via standardized tests, but are more likely to be evident during periods of oral production. However, much of the existing research focuses on the effect of personality traits on language learning strategies, styles and performance measured via standardized tests, which may not represent the optimal context to analyze the existence of meaningful relationships.

This study investigates the oral performance of English as a foreign language (EFL) students engaged in delivering presentations required as part of inquiry-based group projects. Such inquiry-based projects require a high degree of collaboration among team members as they interact with peers in order to create meaningful language and socially construct knowledge (Prince and Felder 2006). Therefore, in addition to the personality dimension, the present research attempts to incorporate both individual and collective orientations of group work (Chow and Law 2005; Dörnyei and Murphey 2003). In doing so, this study extends the existent EFL literature by investigating the effects of personality traits and motivational constructs on student performance in a collaborative oral presentation context.

### Literature Review

#### Five-Factor Model

All human cultures contain lexicon describing individual personality variation, pointing to an intimate connection between language and personality (Dixon 1977). One common method of evaluating these personality characteristics is via the Big Five personality traits/five-factor model (FFM), which is based on lexical descriptors of personality characteristics that group into five domains: Extraversion, Agreeableness, Conscientiousness, Neuroticism and Openness to Experience (McCrae and Costa 1987; Costa and McCrae 1992a). Research has confirmed their universality across languages and cultures (McCrae and Terracciano 2005; Schmitt et al. 2007, 2008), and they consistently appear in factor analyses of personality traits in a diverse range of samples (Costa and McRae 1992b; McCrae and Costa 1997). They are thought to explain inherent differences among individuals and contribute to individual differences that determine a person’s temperament, attitude, cognition, motivation and learning style, which in turn influence one’s academic achievement.

#### Personality and Language Learning

Empirical evidence shows that personality variables influence language learning strategies and thereby affect the quality of language learning (Chen and Hung 2012; Ehrman and Oxford 1995; Oxford and Ehrman 1992). In one of the seminal articles on this topic, Dewaele and Furnham (1999) begin with the premise that producing speech requires the speaker to engage both short- and long-term memory. Notably, psychological studies have generally revealed that extroverts possess advantages in verbal skills, which require short-term recall, while introverts possess advantages when it comes to long-term recall (Dewaele and Furnham 1999; Howarth and Eysenck 1968). Therefore, it is hypothesized that extraverts’ superior verbal processing capacity, which helps them converse with others, stems not only from a superior short term memory, but is also reinforced by enhanced physiological stress resistance and lower levels of social anxiety, which includes oral communication (Dewaele and Furnham 1999). Further, research has shown that, among those with comparable vocabularies, extraverts are able to produce word associations more fluently under conditions of stress and time pressure (Eysenck 1974). Surmising that these factors may affect extraverts’ speech production rather than their entire language learning process, Dewaele and Furnham (1999) assert that:

... some researchers found links between extraversion scores and linguistic variables, depending on the type of linguistic material they used. Whenever extraversion scores were correlated with results from *written tests*, no systematic or significant links appeared ... Significant correlations between extraversion and linguistic variables did appear, however, in *oral communicative speech* ... The nature of the linguistic variable thus appears to affect the possible link with extraversion. Test scores, which are not necessarily good measures of language proficiency, seem less likely to correlate with extraversion scores than fluency measures from oral speech. (p. 521)

They continue this line of reasoning by stating that the more complex the verbal language task, the more likely it will be that a positive correlation between extraversion and linguistic variables will be found, as the ability to process in parallel affords extraverts immense advantages in complex L2 verbal production tasks.

Several prominent studies have been conducted investigating associations between foreign language learning and personality traits such as extraversion. For example, Busch (1982) explored the introversion-extraversion relationship of Japanese junior college $(n=80)$ and adult $(n=105)$ school students and their EFL proficiency. Data was collected via a standardized English proficiency test and oral interviews $(n=45)$, as well as a personality questionnaire. Contrary to the hypothesis that extraverts would demonstrate higher proficiency, a statistical analysis revealed that extraversion was negatively correlated with pronunciation, and that introversion was correlated with higher scores on the grammar and reading sections of the standardized test. However, more extraverted male college students performed better in the oral interviews. In a study conducted in The Intensive English Language Program at Temple University Japan campus, Brown et al. (2001) sampled 320 students and found that more extraverted or socially active students were more motivated learners, and generalize that high proficiency learners can be categorized as: well-balanced in terms of scoring mid-level scores for Thinking Extraversion; emotionally stable with low scores on Inferiority, Feelings and Nervousness; or less instrumentally motivated and less anxious.

Another study conducted on Japanese students came to a similar conclusion (Oya et al. 2004). While they found that individual subcomponents relating to oral production were not significantly correlated with extraversion or neuroticism, they discovered that a global impression score based on raters’ overall considerations of oral performance was positively associated with extraversion. In addition, Ockey (2011) investigated the relationship between two facets of the extraversion domain—assertiveness and self-consciousness—and oral ability of first-year Japanese EFL university students $(n=360)$ using the five factor NEO-PI-R. The analysis revealed assertiveness to be a significant predictor of second language oral ability, specifically communication skills and fluency, for which it explained 3.2 and 3.1%, respectively, of the variance.

#### Motivation and Personality

Motivation is recognized as a key variable in developmental and educational psychology (Dweck 1999; Gardner 1985, 2010) and as an essential element for language learning success (Dörnyei 1998; Dörnyei and Ushioda 2013). Moreover, it has been hypothesized as having a close relationship with a learner’s personality (Dweck and Leggett 1988). Accordingly, Dörnyei and Ushioda (2013) advance that motivation is dependent upon a complex and dynamic interplay between cognitive, contextual, cultural, individual and social factors. Indeed, Dörnyei (1998) reflects that motivation in language learning presents a particularly unique situation:

... the motivational basis of language attainment is not directly comparable to that of the mastery of other subject matters in that knowing an L2 also involves the development of some sort of ’L2 identity’ and the incorporation of elements from the L2 culture (cf. Gardner 1985); thus, in addition to the environmental and cognitive factors normally associated with learning in current educational psychology, L2 motivation also contains featured personality and social dimensions. (p. 118)

Correlation analysis between the Big Five personality traits, measured by the NEO personality inventory, and academic motivation (self-determination theory—intrinsic and extrinsic motivation) and achievement (GPA) was conducted by Komarraju et al. (2009). Conscientious individuals stood out as having the highest intrinsic and extrinsic motivation along with the highest academic performance. Agreeableness and openness were also correlated with achievement. Moreover, regression analyses showed 17% of the variation in intrinsic motivation to be explained by conscientiousness and openness, while 13% of the variance in extrinsic motivation was explained by three personality traits: neuroticism, conscientiousness, and extraversion.

#### Collaboration and Inquiry-Based Motivation

The appeal of collaborative learning stems from the acknowledgement that learning through interaction with peers stimulates meaningful language output and social construction of knowledge (Long 1996; Prince and Felder 2006). Educators realize that such frameworks not only lead to insightful and rewarding educational environments, but also replicate real-world conditions (Aydin and Yildiz 2014). Furthermore, collaborative learning environments are often multifaceted, combining elements such as planning, coordination, teamwork, problem-solving, negotiation, and simulation as students work on projects and presentations (Chu and Kennedy 2011; Stahl et al. 2006; Stoller 2002).

Taking into account the increasing prominence of collaborative inquiry-based learning over the previous two decades, Chow and Law (2005) developed the self-report Collaborative Inquiry-based Project Questionnaire (CIPQ). This instrument aims to investigate motivation in situations where students are likely to be conducting loosely defined projects in teams, rather than individual subject-based tasks. Hence, in addition to extrinsic and intrinsic elements, the CIPQ contains added dimensions to cover group learning and project concepts. The five subscales are: Task, Project Work, Reinforcement, Social Learning and Social Pressure. Task (motivation derived from satisfaction or interest in the activity) and Reinforcement (motivation arising from external rewards or avoidance of negative consequences) factors relate to the traditional intrinsic/extrinsic dichotomy; Project Work connects with the project-related activities inherent in inquiry-based learning contexts conducted in teams; and Social Learning and Social Pressure signify the social aspects of working together in groups and how this shapes collective learning motivation (pp. 72–73). Based on the results of their study and factor analyses using data from students in Hong Kong, the Chinese version of the CIPQ demonstrated validity in terms of gauging motivational constructs in collaborative inquiry-based learning contexts.

### Research Questions

Previous research has identified extraversion as a key personality variable in oral situations (Dewaele and Furnham 1999) and agreeableness and conscientiousness as central determinants of success in group settings (Peeters et al. 2006). While it is generally held that intrinsic motivations are favoured over extrinsic motivations (Flowers 2015; Noels 2009), their importance in Chinese cultural settings has been questioned and motivational factors’ effects on classroom communication are thought to be highly context dependent (Chen et al. 2005; Peng and Woodrow 2010). For example, Peng and Woodrow (2010) find evidence of both external regulation and intrinsic motivation influencing confidence to communicate in English. Preparing and presenting effective presentations requires communication abilities extending beyond the verbal domain; therefore, affective factors conducive to interacting with others in groups are potentially influential variables. To date, there has been little research conducted on the relationship between personality and motivation in inquiry-based learning contexts where collaborative presentations were the means of assessment. Consequently, the present study attempts to extend the scope of the existing literature by examining personality traits and motivational constructs within the context of collaborative presentations. The following research questions are considered:

1. Which Big Five Inventory (BFI) personality traits are associated with collaborative oral presentation performance?
2. Which CIPQ motivational constructs are associated with collaborative oral presentation performance?
3. Are BFI personality traits associated with CIPQ motivational constructs?

## Materials and Methods

### Context

During this period of unprecedented globalization and rapid technological change, many Taiwanese universities are following the global trend of striving to teach students relevant content and skills through the implementation of English for specific purposes (ESP) programs. A primary aim of these programs is to develop language skills necessary for success in the job market and students’ future careers (Chien and Hsu 2010; Tsai 2010). Workplace presentations to internal and/or external audiences are commonplace in many firms; as such, presentations have become a popular means of both instructing and assessing oral communication skills. However, presentations are often challenging for EFL students, as their needs with respect to structure and formality are not met by the content offered in most general EFL classrooms (Ferris 1998; Tsai 2010). EFL students enrolled in an ESP program at a university in northern Taiwan participated in this study, where collaborative oral presentation scores served as the dependent variable (DV).

**Fig. 1:**
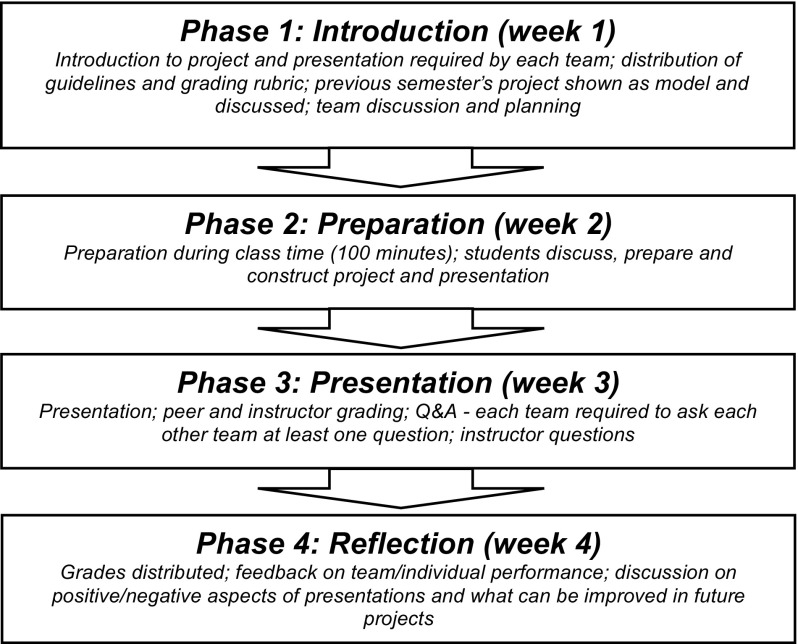
Phases of collaborative inquiry-based project and presentation

### Participants

Of the 329 students enrolled in the 12 classes selected, 257 (78.12%) agreed to participate in the study. The sample included students registered in the spring (five classes) and fall (four classes) semesters of 2015, and the spring (three classes) semester of 2016. The participants were comprised of Mandarin-speaking undergraduate students from six different colleges—Law, Business, Humanities, Social Sciences, Engineering, and Public Affairs—enrolled in one of three ESP courses: Business Case Studies (six classes), Workplace English (one class) or Culture and Tourism (five classes). Data on gender $(\hbox {female}=168, \hbox {male}=89)$, age $(M=20.38, SD=1.63)$ and average Test of English for International Communication (TOEIC) scores $(M=740, SD=100)$ of the participating students was collected.

### Collaborative Projects, Presentations and Ratings

The entire project lasted for four weeks; the timetable is displayed in Fig. 1. Collaborative oral presentations lasted for 10–12 min and were conducted by teams of 3–6 participants, allowing students an average speaking time of approximately 2–4 min each. With a restricted speaking time, coordination among team members was crucial to maintain smooth transitions between members and ensure an effective presentation. All students were required to be involved in the presentation and grading process. Three different presentation topics were employed for the three ESP courses:

- *Business Case Studies* presentations required developing persuasive presentations based on stocks, bonds and other investments teams selected following their research.
- *Workplace English* teams presented on domestic or international corporations covering aspects such as the founder, history, mission, products, services, locations, strategy, advertising.
- *Culture and Tourism* students delivered presentations on a local food reporting on the history, culture, ingredients, recipes, taste, texture, aroma, and famous vendors.

**Fig. 2:**
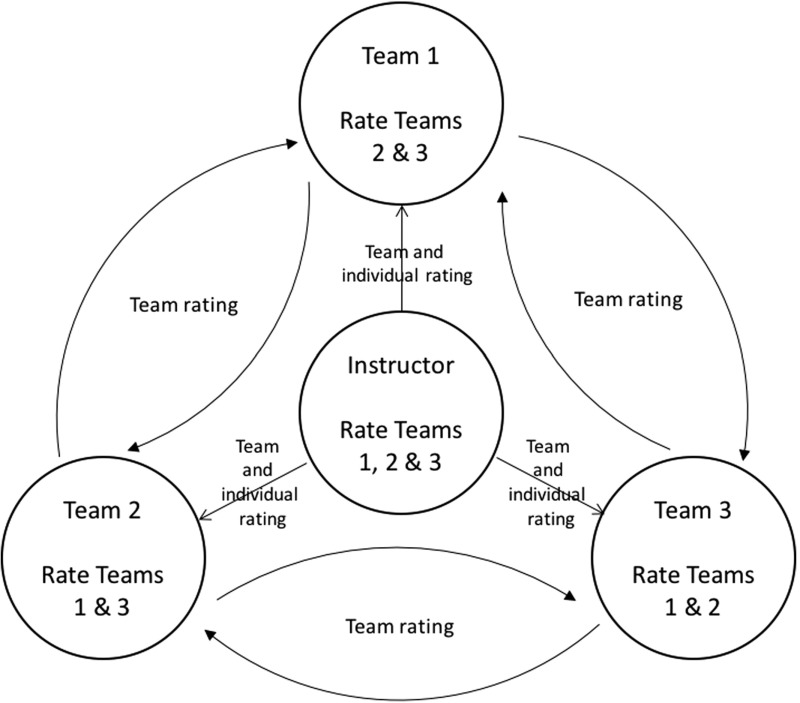
Presentation scoring system

Peer rating has been viewed as a method to include learners in the evaluation process, foster fair and objective assessment and promote deeper learning (Otoshi and Heffernen 2008; Peng 2010; Saito 2008). In fact, research has promoted peer rating as a particularly valid approach to assessment for the rating of oral scores (Cheng and Warren 2005; p. 12) and presentation performance (De Grez et al. 2012), and it can be argued that the employment of group peer rating is a means to reduce bias in peer rating (Aryadoust 2016). As such, team peer ratings combined with instructor’s individual and team ratings are utilized in this study. Presentation scores were calculated as follows: i) the instructor rated each student and team, and ii) each team rated each other team. Students’ scores on a scale from 1 to 100 for the presentation were calculated as an average of the three ratings. A diagram depicting the presentation scoring method (reduced to three teams and instructor for simplicity) is displayed in Fig. 2. Inter-rater agreement for the team scores rated by the instructor and each team ranged from 0.62 and 0.81, indicating substantial agreement (Landis and Koch 1977).

Ratings for presentations were based on a scoring rubric (see Appendix), which was decided upon by the participating students and posted on the class e-learning website so that each team could use it as a guide while preparing their presentation.

**Table 1:** Total and participant presentation score means and standard deviations

Semester/class	Total $(N=329)$	Presentation score (total class)		Participants $(n=257)$	Presentation score (participants)	
		M	SD		M	SD
*Spring 2015*						
Culture and tourism	31	79.39	5.66	27	78.07	2.67
Culture and tourism	30	78.03	2.88	24	78.21	5.78
Business case studies	29	82.0	3.17	27	82.26	4.1
Business case studies	28	82.14	4.26	26	81.02	2.68
Workplace English	15	82.93	2.12	10	82.30	2.75
*Fall 2015*						
Culture and tourism	28	78.25	3.01	23	78.7	3.38
Culture and tourism	30	78.11	3.05	28	80.43	3.23
Business case studies	29	82.28	2.39	19	81.0	4.11
Business case studies	29	77.36	3.4	25	81.04	4.44
*Spring 2016*						
Culture and tourism	27	78.63	1.9	12	80.67	3.7
Business case studies	24	79.42	2.04	17	81.71	2.11
Business case studies	29	78.97	1.52	19	82.26	2.86

- Presentation (40%)—organization/structure, clarity of visual slides, clarity of message, relevance of material
- Delivery (50%)—appropriate language, pronunciation/clarity of voice/fluency, eye contact/body language, enthusiasm/presence, time management/flow/teamwork
- Question and answer (10%)—includes asking and responding to questions

A variety of approaches can be found regarding measurement of language achievement and performance in the literature. Accordingly, Oya et al. (2004) discuss some of the contradictions found within the oral speech production measures of other studies and find no significant correlations in their own study regarding any sub measures (Fluency, Accuracy, Complexity) of oral proficiency; however, they do find a significant positive correlation between global impression (overall measure of proficiency) and extraversion (p. 848). Due to the collaborative nature of the presentations under investigation and the integration of peer (team) and instructor (individual and team) assessment in the present study, a composite rating (Score) incorporating linguistic (speech), meta-linguistic (delivery) and paralinguistic (verbal and nonverbal) elements as well as visual communication was preferred for use as the DV. By employing an aggregate achievement measure (e.g., Woodrow 2006; Patri 2002), the authors of the current study attempt to provide a holistic interpretation of collaborative oral presentation performance. Accordingly, the achievement measure permits the subjectivity required for the assessment of the dynamic, interactive and interrelated social relationships influencing both the cohesion and functioning of team participants and their interactions with the audience in this collaborative oral presentation setting (Clément et al. 1994; Underhill 1987).

Presentation scores for each class and for those who participated in the study are recorded in Table 1. Scores for participants in the study ranged from 66 to 89 with an average of 80.48 and a standard deviation of 3.9. An omnibus *F*-test following Analysis of variance (ANOVA) found statistically significant differences between presentations scores for the three different classes: $F(2,256)=9.23, p<0.001, \upeta ^{2}=0.07$. A post hoc Bonferroni test found a statistically significant difference between presentation scores for Culture and Tourism classes and Business Case Studies classes $(\hbox {M}_{\mathrm{diff}}=1.93, \hbox {SE}=0.48, p<0.001)$.

### Instruments

*BFI: *Personality traits were measured via the publically available Chinese version of the BFI-44 (Benet-Martinez and John 1998; John et al. 1991, 2008) and distributed using GoogleForms during class time before the presentation projects began. This instrument is commonly used in research, shows strong psychometric properties and convergence with other five-factor models of personality (Carciofo et al. 2016). The 44 items were assessed on 5-point Likert scales, where the Cronbach’s alpha for each domain: Extraversion (0.77), Agreeableness (0.73), Conscientiousness (0.76), Neuroticism (0.70) and Openness to Experience (0.73) presented acceptable internal consistency. Table 2 displays the inter-scale correlations of this instrument.

**Table 2:** BFI-44 inter-scale correlations

Scale	1.	2.	3.	4.
1. Extraversion	–			
2. Agreeableness	.07	–		
3. Conscientiousness	.14*	.34**	–	
4. Neuroticism	$-$ .22**	$-$ .30**	$-$ .37**	–
5. Openness to Experience	.24**	.21**	.20**	$-$ .04

*p value < .05; **p value < .01

*CIPQ: *The 20-item CIPQ uses 7-point Likert scales, and has been employed in numerous studies in east Asian contexts showing good internal consistency (Chow and Law 2005; Lam 2009; Flowers 2015). The questionnaire was distributed in electronic form during class time at the end of the presentation projects using GoogleForms. Cronbach’s alpha figures for internal consistency were: Task (0.72), Reinforcement (0.67), Project Work (0.75), Social Learning (0.77) and Social Pressure (0.76). Table 3 shows the inter-scale correlations for the CIPQ.

**Table 3:** CIPQ inter-scale correlations

Item	1.	2.	3.	4.
1. Task	–			
2. Reinforcement	.40**	–		
3. Project Work	.77**	.39**	–	
4. Social Learning	.73**	.43**	.86**	–
5. Social Pressure	.44**	.64**	.50**	.63**

*p value < .05; **p value < .01

**Table 4:** Variable means and standard deviations

	Total ($n=257$)		Female ($n=158$)		Male ($n=89$)		High TOEIC ($n=121$)		Low TOEIC ($n=116$)	
	*M*	*SD*	*M*	*SD*	*M*	*SD*	*M*	*SD*	*M*	*SD*
Score	80.48	3.90	80.90	3.50	79.67	4.47	81.62	3.21	79.32	4.33
TOEIC	740	100	745	101	730	98	817	66	659	55
Extra	3.17	0.61	3.17	0.60	3.16	0.63	3.19	0.60	3.14	0.64
Agree	3.58	0.53	3.60	0.55	3.54	0.48	3.59	0.51	3.58	0.55
Consc	3.21	0.55	3.18	0.56	3.26	0.53	3.21	0.59	3.22	0.52
Neuro	3.01	0.55	3.06	0.57	2.91	0.50	3.02	0.56	3.01	0.54
Open	3.32	0.56	3.35	0.60	3.28	0.46	3.36	0.54	3.28	0.58
Task	5.15	1.20	5.16	1.25	5.11	1.08	5.18	1.27	5.09	1.18
Reinforce	3.36	1.20	3.31	1.26	3.47	1.07	3.31	1.06	3.37	1.31
Project	4.88	1.18	4.91	1.22	4.82	1.09	4.95	1.24	4.82	1.14
SocLearn	4.76	1.18	4.82	1.23	4.65	1.09	4.84	1.21	4.73	1.17
SocPress	4.00	1.23	3.95	1.28	4.10	1.14	3.93	1.22	4.07	1.26

High TOEIC and low TOEIC do not add up to total as a result of missing values

## Results

### Initial Data Analysis

The results reveal that the quality of presentations and scores awarded were relatively high across all groups of students examined. Means and standard deviations for the entire sample, female and male participants and high and low English ability are shown in Table 4. Initial data analysis to examine differences between BFI and CIPQ scales and presentation scores was conducted using independent t-tests with gender and high and low TOEIC scores (High level > 720; Low level $\le$ 720) as independent variables. For gender, significant differences were found for neuroticism (female $M=3.06, SD=0.57$; male $M=2.91, SD=0.50; t(255)=2.16, p<0.05$) and Score (female $M=80.9, SD=3.5$; male $M=79.67, SD=4.47; t(255)=2.43, p<0.05$); for high and low TOEIC scores, significant differences did not arise for any of the BFI or CIPQ scales, but did for Score (Low level $M=79.32, SD=4.33$; High level $M=81.62, SD=3.21; t(235)=-4.66, p<0.001$).

The Extraversion scale of the BFI is comprised of eight items measuring different aspects of an extraverted/introverted personality: “Is talkative”, “Is reserved”, “Is full of energy”, “Generates a lot of enthusiasm”, “Tends to be quiet”, “Has an assertive personality”, “Is sometimes shy, inhibited”, “Is outgoing, sociable”. For convenience of understanding, the items are termed: Talkative, Unreserved, Energetic, Enthusiastic, Vocal, Assertive, Uninhibited, Outgoing. Items Unreserved, Vocal and Uninhibited were reversed for analysis. The means and standard deviations are displayed in Table 5.

**Table 5:** Extraversion item means and standard deviations

Item	Total ($n=257$)		Female ($n=158$)		Male ($n=89$)		High TOEIC ($n=121$)		Low TOEIC ($n=116$)	
	*M*	*SD*	*M*	*SD*	*M*	*SD*	*M*	*SD*	*M*	*SD*
Talkative	3.29	1.02	3.32	1.02	3.24	1.01	3.33	1.04	3.22	1.04
Unreserved	3.10	1.03	3.13	1.00	3.06	1.07	3.09	0.99	3.14	1.08
Energetic	3.56	0.83	3.57	0.82	3.55	0.84	3.54	0.81	3.59	0.85
Enthusiastic	3.33	0.90	3.35	0.90	3.30	0.91	3.38	0.90	3.26	0.93
Vocal	3.19	1.07	3.23	1.02	3.11	1.16	3.19	0.98	3.20	1.20
Assertive	2.90	0.95	2.86	0.98	2.98	0.89	2.90	0.92	2.90	1.01
Uninhibited	2.68	0.98	2.63	0.98	2.78	0.97	2.69	0.90	2.65	1.05
Outgoing	3.26	1.02	3.26	1.05	3.27	0.96	3.35	1.04	3.15	1.02

### Correlation Analysis

#### Score and Personality Traits

Analyzing the full sample, the variable Score was only correlated with the Extraversion personality trait ($r=0.25, p<0.01$). Items from the Delivery subcomponent of Score relating to appropriate language ($r=0.14, p<0.05$), pronunciation/clarity of voice/fluency ($r=0.25, p<0.01$), eye contact/body language ($r=0.24, p<0.01$), enthusiasm/presence ($r=0.26, p<0.01$), time management/flow/teamwork ($r=0.18, p<0.01$) also correlated with Extraversion. TOEIC correlated with Extraversion ($\hbox {r}=0.32, p<0.01$) and Openness to Experience ($\hbox {r}=0.19, p<0.01$). At the item level, analysis showed Extraversion items Talkative ($r=0.16, p<0.01$), Energetic ($r=0.13, p<0.05$), Enthusiastic ($r=0.19, p<0.01$), Assertive ($r=0.21, p<0.01$) and Outgoing ($r=0.27, p<0.01$) correlated with Score, while Score correlated with Extraversion for both females ($r=0.24, p<0.01$) and males ($r=0.27, p<0.01$). For females, correlations were recorded for Enthusiastic ($r=0.19, p<0.05$), Assertive ($r=0.26, p<0.01$), and Outgoing ($r=0.23, p<0.01$); for males, correlations were registered for Talkative ($r=0.25, p<0.05$) and Outgoing ($r=0.36, p<0.01$). Further, analyzing the data based on TOEIC scores, there was no correlation between Score and high level students for extraversion, but there was between score and low level students ($r=0.37, p<0.01$). At the item level, Assertive ($r=0.23, p<0.05$) correlated with Score for higher level students, while for lower level students, Talkative ($r=0.28, p<0.01$), Reserved ($r=0.22, p<0.05$), Enthusiastic ($r=0.25, p<0.01$), Quiet ($r=0.26, p<0.01$), Assertive ($r=0.20, p<0.05$), and Outgoing ($r=0.36, p<0.01$) all correlated with Score.

#### Score and Motivation

Using the full dataset, Score correlated with the motivational construct Project Work ($r=0.17, p<0.05$). Project work item “I participate in project work because it is fun” ($r=0.15, p<0.05$), Item “Because it is important to do project work” ($r=0.17, p<0.01$) and item “Because participating in project work can help my academic learning” ($r=0.14, p<0.05$) correlated with Score at the item level. For convenience of understanding, these items will subsequently be termed: Fun, Important, Academic and Courage. For females, significant correlations were found for Score and Project Work ($r=0.19, p<0.05$), as well as Score and Social Learning ($r=0.16, p<0.05$). At the item level, the results show correlations between Score and Project Work items Important ($r=0.25, p<0.01$) and Academic ($r=0.16, p<0.05$); and for Social Learning item “Because learning in a group allows me to have more courage to investigate more complex topics” ($r=0.20, p<0.05$). Motivation did not correlate with any domains for males, nor with high or low TOEIC scores.

#### Personality Traits and Motivation

To investigate the association between motivation and personality traits, correlations between BFI and CIPQ domains were calculated. Regarding the scales on the two questionnaires, significant correlations were found between Task and Extraversion ($r=0.20, p<0.01$) and Project Work and Extraversion ($r=0.14, p<0.05$). At the item level, Task correlated with Extraversion items Talkative ($r=0.23, p<0.01$), Energetic ($r=0.20, p<0.01$), Enthusiastic ($r=0.24, p<0.01$) and Outgoing ($r=0.24, p<0.01$), and Project work with Talkative ($r=0.14, p<0.05$), Energetic ($r=0.17, p<0.01$), Enthusiastic ($r=0.15, p<0.05$) and Outgoing ($r=0.13, p<0.05$). With regard to females, Task correlated with Extraversion items Talkative ($r=0.21, p<0.01$), Energetic ($r=0.26, p<0.01$), Enthusiastic ($r=0.25, p<0.01$) and Outgoing ($r=0.26, p<0.01$), while Project Work correlated with Enthusiastic ($r=0.16, p<0.05$) and Assertive ($r=0.16, p<0.05$). For males, correlations were found between Task and items Talkative ($r=0.27, p<0.05$) and Enthusiastic ($r=0.22, p<0.05$), while none were uncovered for Project Work. Furthermore, after analyzing the results based on TOEIC scores, higher level English students displayed correlations between Task and Extraversion items Talkative ($r=0.21, p<0.05$), Energetic ($r=0.21, p<0.05$), Enthusiastic ($r=0.21, p<0.05$) and Outgoing ($r=0.24, p<0.05$), and Project Work and Assertive ($r=0.20, p<0.05$). Finally, for lower level English students, correlations were noted between Task and Extraversion items Talkative ($r=0.23, p<0.05$), Energetic ($r=0.19, p<0.05$) and Enthusiastic ($r=0.25, p<0.01$) and Project Work and items Talkative ($r=0.22, p<0.05$), Energetic ($r=0.22, p<0.05$), Enthusiastic ($r=0.24, p<0.05$) and Outgoing ($r=0.21, p<0.05$). A significant correlation was also observed for Social Pressure and Conscientiousness ($r=-0.17, p<0.01$).

### Regression Analysis

To gain further understanding of the data and identify which explanatory variables made contributions in explaining the DV Score, regression analysis was employed. In doing so, it was possible to test different models and detect the amount of variance explained by the selected explanatory variables.

#### Score and Personality Traits and Motivation

Factors with significant correlations with the DV Score were selected as predictor variables and sequentially entered into regression equations. Results of the six ensuing regression models are displayed in Table 6. Extraversion was the sole selected personality variable, explaining 6% of the variation; motivational variables Project Work and Social Pressure were selected, explaining a combined 4% of the variance. Following this, personality and motivational variables were included together, explaining 8% of the variation. When TOEIC was selected as an additional explanatory variable along with personality and the motivation predictor variable, the model accounted for 15% of the variability in Score. The model including gender with the aforementioned predictors led to a model explaining 16% of the variability in Score, suggesting that the inclusion of gender added little explanatory power to the regression model. Finally, entering a variable to account for differences over the types of classes improved the model’s explanatory power to 18%.

**Table 6:** Regression coefficients for DV Score

Model	Predictor variables	*Beta*	*t*	*df*	*adj r* $^{2}$	*F*
Model 1	Extraversion	1.59	4.09***	255	0.06	16.73***
Model 2	Project	0.82	3.44**			
	Social Pressure	$-$ 0.53	$-$ 2.34*	236	0.04	6.73**
Model 3	Extraversion	1.34	3.46**			
	Project	0.68	2.88**			
	Social Pressure	$-$ 0.46	$-$ 2.06*	235	0.08	8.3***
Model 4	Extraversion	1.25	3.19***			
	Project	0.54	2.27*			
	Social Pressure	$-$ 0.34	$-$ 1.50			
	TOEIC	0.01	4.18***	216	0.15	10.72***
Model 5	Extraversion	0.20	3.20**			
	Project	0.16	2.20*			
	Social Pressure	$-$ 0.10	$-$ 1.34			
	TOEIC	0.26	4.06***			
	Gender	$-$ 0.10	$-$ 1.65	215	0.16	9.18***
Model 6	Extraversion	0.22	3.45**			
	Project	0.23	1.83			
	Social Pressure	$-$ 0.14	$-$ 1.16			
	TOEIC	0.23	3.71***			
	Class	0.18	2.83**	215	0.18	10.3***

Extraversion, Project and Social Pressure were confirmed as predictor variables by way of stepwise regression procedures

*p value < .05; **p value < .01; ***p value < .001

At the item level, stepwise regression analysis selected items Assertive and Outgoing (${\textit{Beta}}=0.25, t=2.90, p<0.01$ and ${\textit{Beta}}=0.23, t=4.12, p<0.001, {\textit{df}}=254, F=14.67, p<0.001, {\textit{adj r}}^{2}=0.10$) from the Extraversion domain. For Project Work, the item Important was selected as the predictor variable (${\textit{Beta}}=0.17, t=2.68, p<0.01, {\textit{df}}=237, F=7.18, p<0.01, {\textit{adj r}}^{2}=0.03$) explaining 3% of the variation in Score. Social Pressure, items “Because if I don’t participate, my groupmates will blame me” and “Because I don’t want to be perceived as a burden of my groupmates” were selected (${\textit{Beta}}=-0.18, t=-2.70, p<0.01$ and ${\textit{Beta}}=0.16, t=2.51, p<0.05, {\textit{df}}=237, F=5.75, p<0.001, {\textit{adj r}}^{2}=0.04$) and together explained 4% of the variation in Score.

#### Personality Traits and Motivation

Additional regressions were performed using motivational variables as the DVs and entering personality variables as predictors based on the significant correlations revealed above. Table 7 shows the results of these variable selections. Task work was best explained by Extraversion, explaining 4% of the variation; Project Work by Extraversion, explaining 2%; and Social Pressure by Conscientiousness, explaining 2%.

**Table 7:** Regression coefficients for motivational DVs

Dependent variables	Predictor variables	*Beta*	*t*	*df*	*adj r* $^{2}$	*F*
Task	Extraversion	0.39	3.18**	237	0.04	10.09**
Project	Extraversion	0.27	2.21*	237	0.02	4.9*
Social Pressure	Conscientious	$-$ 0.38	$-$ 2.62**	237	0.02	6.85**

Extraversion and Conscientiousness were confirmed as predictor variables by way of stepwise regression procedures

*p value < .05; **p value < .01; ***p value < .001

At the item level, stepwise regression analysis selected Extraversion items Talkative and Enthusiastic (${\textit{Beta}}=0.15, t=2.1, p<0.05$ and ${\textit{Beta}}=0.17, t=2.3, p<0.05, {\textit{df}}=236, F=9.43, p<0.001, {\textit{adj r}}^{2}=0.07$) for Task; Extraversion item Energetic (${\textit{Beta}}=0.17, t=2.68, p<0.01, {\textit{df}}=23, F=7.16, p<0.01, {\textit{adj r}}^{2}=0.03$) for Project Work; and Conscientiousness item “Tends to be disorganized” (${\textit{Beta}}=-0.21, t=-3.38, p<0.01, {\textit{df}}=237, F=11.41, p<0.01, {\textit{adj r}}^{2}=0.04$) for Social Pressure.

## Discussion

The purpose of this study was to examine the influence of personality and motivation on performance in a collaborative oral presentation context. Performance was measured as a composite of instructor (individual and team) and peer (team) ratings. A notable finding was that Extraversion was the only personality trait significantly correlated with presentation performance. A closer look at the delivery aspect of the presentations showed significantly positive correlations between all subsections and Extraversion, with the verbal component ‘pronunciation/clarity of voice/fluency’ and nonverbal elements ‘eye contact/body language’ and ‘enthusiasm/presence’ holding higher associations. Furthermore, within the Extraversion domain, five items displayed significant correlations, indicating that talkative, energetic, enthusiastic, assertive and outgoing/sociable students were more likely to perform well in collaborative oral presentations. In addition, regression analysis revealed extraversion explained 6% of the variation in oral presentation performance. Therefore, as noted in past research, it appears that extraverts do possess the upper hand when it comes to oral presentation performance, broadly echoing the findings of aforementioned studies (for example Brown et al. 2001; Ockey 2011) and potentially supporting Dewaele and Furnham’s (1999) contention that extraverts enjoy an advantage in oral production, likely owing to their enhanced ability to cope with higher levels of stress as a result of lower arousal levels.

Further examination of the data showed that lower ability students’ performance in the oral presentation positively correlated with almost all of the extraversion items, while higher ability students’ scores correlated primarily with being assertive. Regression analysis tended to support this contention with TOEIC revealed as a significant predictor of presentation performance. Therefore, extraversion traits manifest to a greater degree in the achievement of students with lower English ability. These results suggest that lower ability students benefit from the presence of the extraversion characteristics associated with collaborative oral presentation scenarios, where an outgoing personality can compensate for insufficient language ability, particularly when this personality trait results in the ability to overcome a lack of confidence. With respect to associations corresponding to gender and oral presentation performance, correlations for female students related to factors such as enthusiasm, assertive personality and outgoing/sociable personality types, while those for males correlated with students who were talkative and outgoing/sociable. Additionally, females appreciated the social aspect of learning, feeling that it afforded them courage to extend themselves to study complex topics. Nevertheless, inclusion of gender in regression models did not significantly improve their predictive power.

Multiple regression analysis also uncovered that motivation derived from the project work aspect of the assignment was associated with performance in the collaborative oral presentation. Project work motivation relates to the positive values attached to the fun, importance and academic aspect of this type of collaborative activity. When contrasted with the lack of significance of Task motivation, this implies that “students considered project work as activities distinct from general school work” and “possibly [reflects] their identification [with] the inquiry nature of project work” (Chow and Law 2005, p. 73). Put another way, motivation arising from the inquiry-based project nature of the assignment prevailed over that of traditional intrinsic motivation.

Furthermore, the selection and significance of Social pressure as a predictor of collaborative oral presentation performance adds an interesting contrast to this consideration of intrinsic motivation and shares similarity with the results of Flowers (2015) who found friendship and reciprocity as motivators for Japanese tertiary students engaged in a computer-supported collaborative learning context. However, the results of the present study lend themselves to a slightly different interpretation. Social pressure here represents two forces working in opposite directions, i.e., unease about being blamed acting as a negative stimulus and concern over the maintenance of one’s reputation as a positive influence. Regarding the first item, the field of positive psychology points to a possible explanation in that environments where constructive conditions are met, such as the presence of empathy, lead to optimal functioning of people and teams (Gable and Haidt 2005; Linley et al. 2006). Similarly, a positive attitude towards language learning has been advanced as a facilitator of the learning process (Gardner 2010; MacIntyre and Mercer 2014). The second item finds support from research indicating the predominance of external motivators such as instrumental and required motivation in Chinese cultural settings (Chen et al. 2005; Peng and Woodrow 2010). In addition, the significant negative association of Conscientiousness with Social Pressure suggests that less diligent students may come under social pressure from team members seeking positive team performance.

Practical implications for educators relate to setting situations conducive to lowering anxiety levels, permitting in-class preparation and coaching, and allowing time for rehearsal may be helpful strategies for those who require them. Along with creating friendly supportive classroom environments, promoting the importance of instructors knowing who their students are, gauging their attitudes towards oral language production, and understanding potential reasons for a lack of willingness to engage in oral speaking production have been revealed as crucial in motivating students to speak (Lee 2016; Tsiplakides and Keramida 2009). It is worthy to note that all of these are particularly important in Confucian Heritage Cultures, where students have been found to be especially influenced by affective variables such as anxiety and nervousness in foreign language classrooms (Lee 2016; Woodrow 2006).

Preparing and delivering presentations requires both presentation-specific and higher-order thinking and paralinguistic capabilities. For the former, developing relevant creative, heuristic, metacognitive, problem-solving, and teamwork competencies can be incorporated into classroom undertakings as students prepare, organize, coordinate, communicate, negotiate and prepare to deliver their presentations (Chou 2011; O’malley et al. 1985). With regard to the latter, offering paralinguistic training such as voice control, pitch and prosody, along with body movement and gesture coaching can increase student confidence while delivering their presentations (Bankowski 2010; Morley 1991). Additionally, research suggests that student inclusion in presentation goal setting and achievement criteria can be employed to motivate student participation and performance in presentations (Al-Issa and Al-Qubtan 2010; Otoshi and Heffernen 2008). As such, incorporating goal setting as a pre-presentation classroom activity may help bridge the gap between teachers’ and students’ differing perceptions of the purpose of oral presentations, and facilitate better understanding of how students may achieve desired outcomes for presentations.

## Conclusion

The present study offers evidence for the role personality traits and collaborative inquiry-based motivation play in explaining achievement in oral presentations conducted by teams. Specifically, it points to the importance of the personality trait extraversion, along with project work and social pressure motivational factors. Furthermore, indication of the personality characteristics extraversion and conscientiousness in motivational constructs relating to inquiry-based collaborative learning is provided. These findings provide support for the argument that extraverts are likely to perform better in spoken tasks, potentially pointing to their superior verbal processing capacity and lower language speaking anxiety as they interact with peers in collaborative projects and engage in oral presentations. In addition, it highlights the importance of both intrinsic and extrinsic motivational factors concurrently playing a role in collaborative inquiry-based projects as teams of students prepare and deliver oral presentations.

While the current study extends the existing literature on individual differences in affective factors within an EFL context, it is subject to several limitations. First, it almost exclusively reports on presentation performance of mid- to high-level English ability university students. Research conducted on groups including a greater range of learners according to English ability may reveal different outcomes. Additionally, neither the academic discourse socialization of the students (Ho 2011; Zappa-Hollman 2007) nor record of accuracy and lexical density are considered in light of the speed-accuracy trade off identified in previous literature (Dewaele and Furnham 1999; MacKay 1982). The use of a composite variable incorporating both verbal and non-verbal communication for presentation performance may be viewed as both a weakness and a strength. One the one hand, it does not solely focus on the oral speech production of the learners, yet it does include valuable information on non-verbal aspects of communication crucial to delivering effective presentations. Accordingly, the authors acknowledge that measures based solely on verbal assessment may offer different results to those presented in this research. Investigating the hypothesis that extraverts prefer fluency while introverts favor lexical richness, further examination of students’ academic discourse in EFL oral presentations, and consideration of different measures of communication ability offer potential directions for future research.

## Appendix: Presentation Grading Rubric

**Table:**

Grading category	Group 1	Group 2	Group 3	Group 4	Group 5	Group 6	Group 7	Group 8
*Content (40%)*								
Organization/structure								
Slides (PowerPoint)								
Message								
Relevance								
*Delivery (50%)*								
Language								
Pronunciation/voice/fluency								
Eye contact/body language								
Enthusiasm/presence								
Time management/flow								
*Q&A (10%)*								
*Total (100%)*								

Total Score $$> 90 = \hbox {excellent}$$, 80 – $$89 = \hbox {very good}$$, 70 – $$79 = \hbox {good}$$, 60 – $$\hbox {69} = \hbox {adequate}, < 60 = \hbox {fail}$$

**Table:**

Category	Points		
	10-8	7-5	4-0
*Presentation (40%)*			
Organization/structure	Excellent, logical structure and organization make the presentation easy to follow and understand	Adequate structure and organization to follow and understand presentation; some revisions may help understanding	Structure and organization needed to improve the logical flow of the presentation and make it easier to understand
Slides (PowerPoint)	Excellent slides; correct font; easy to read; excellent visuals/pictures	Slides adequate; sufficiently readable; acceptable visuals/pictures	Slides need to be made easier to read; visual images need improvement
Message	Message is explained very clearly and informatively; expressed creatively	Message expressed reasonably clearly; delivered with some degree of creativity	Message unclear; often not explained clearly and without creativity
Relevance	All information is relevant and presenters show excellent knowledge of the topics	Content is mostly relevant and presenters have adequate background knowledge	Some content is irrelevant; appears that presenters may not have adequate background knowledge
*Delivery (50%)*			
Language	Excellent use of target language (English); appropriate vocabulary; absence of grammatical errors	Mostly used target language (English); grammar and vocabulary used adequately to express message; relatively few errors	Lack of target language (English) use; requires more appropriate vocabulary; obvious grammatical mistakes
Pronunciation/voice/fluency	Spoken with excellent articulation, fluency and clarity; appropriate volume for the audience to easily follow	Good articulation, fluency and clear speech; usually at adequate volume	Difficult to follow due to poor articulation, pronunciation and fluency; volume sometimes not at appropriate level

**Table:**

Eye contact/body language	Excellent eye contact with the audience at all times; use effective and confident body language and gestures	Good eye contact; reasonably confident; acceptable body language and gestures	Lack of eye contact; lacking confidence; body language and gestures need improvement
Enthusiasm/presence	Presented enthusiastically by passionate presenters who connect with the audience	Some enthusiasm shown by presenters who appear to have some passion and attempt to connect with audience	Little enthusiasm by presenters who appear to lack passion and struggle to connect with audience
Time management/flow	Excellent time management; clearly shows the presenters have rehearsed well; equally shared time with excellent, smooth transitions between presenters	Acceptable time management; some evidence of rehearsal; reasonably equally shared time and adequate transitions	Poor time management; no evidence of rehearsal; unequal time distribution and poor transitions
*Q&Q (10%)*			
Q&A	Ask excellent, well-thought questions to test understanding of the topic; answer any questions accurately without hesitation	Good questions asked and adequately attempted to answer questions	Lack of thought in questions; irrelevant or incorrect questions; poorly answered questions

Score $$> 90 = \hbox {excellent}$$, 80 – $$89 = \hbox {very good}$$, 70 – $$79 = \hbox {good}$$, 60 – $$69 = \hbox {adequate}, < 60 = \hbox {fail}$$
